# Spike protein of SARS-CoV-2 Omicron variant: An *in-silico* study evaluating spike interactions and immune evasion

**DOI:** 10.3389/fpubh.2022.1052241

**Published:** 2022-11-29

**Authors:** Jose A. Jimenez Ruiz, Cecilia Lopez Ramirez, Jose Luis Lopez-Campos

**Affiliations:** ^1^Research Group on Electronic Technology and Industrial Computing (TIC-150) at the University of Seville, Seville, Spain; ^2^Unidad Médico-Quirúrgica de Enfermedades Respiratorias, Instituto de Biomedicina de Sevilla (IBiS), Hospital Universitario Virgen del Rocío/Universidad de Sevilla, Seville, Spain; ^3^Centro de Investigación Biomédica en Red de Enfermedades Respiratorias (CIBERES), Instituto de Salud Carlos III, Madrid, Spain

**Keywords:** SARS-CoV-2, COVID-19, immune evasion, *in-silico*, coronavirus

## Abstract

**Background:**

The fundamentals of the infectivity and immune evasion of the SARS-CoV-2 Omicron variant are not yet fully understood. Here, we carried out an *in-silico* study analyzing the spike protein, the protein electrostatic potential, and the potential immune evasion.

**Methods:**

The analysis was based on the structure of the spike protein from two SARS-CoV-2 variants, the original Wuhan and the Botswana (Omicron). The full-length genome sequences and protein sequences were obtained from databanks. The interaction of the spike proteins with the human Angiotensin Converting Enzyme 2 (ACE2) receptor was evaluated through the open-source software. The Immune Epitope Database was used to analyze the potential immune evasion of the viruses.

**Results:**

Our data show that the Omicron spike protein resulted in 37 amino acid changes. The physicochemical properties of the spike had changed, and the electrostatic potentials differed between both variants. This resulted in a decrease in protein interactions, which does not establish a greater interaction with the ACE2 receptor. These changes compromise key receptor-binding motif residues in the SARS-CoV-2 spike protein that interact with neutralizing antibodies and ACE2.

**Conclusions:**

These mutations appear to confer enhanced properties of infectivity. The Omicron variant appears to be more effective at evading immune responses.

## Introduction

The successive variants of SARS-CoV-2 that have appeared have posed a challenge for the scientific community, constituting a source of uncertainty for clinicians in charge of patient care and a challenge for public health preventive measures (1–3). The possible changes in the therapeutic responses of the available treatments, as well as the possible impact on the efficacy of the vaccines, have made it necessary to identify and characterize, as effectively as possible, each appearance of a new variant in order to coordinate a suitable health response.

Over recent months, the appearance of a new variant in South Africa has contributed to the further expansion of the virus worldwide, with the appearance of a new wave of cases, with obvious clinical consequences (4, 5). This recent variant, named SARS-CoV-2 Omicron, encodes 37 amino acid substitutions in the spike protein, 15 of which are in the receptor-binding domain (RBD). Based on our study of physicochemical interaction of the Omicron variant spike proteins with the human ACE2 receptor, we have seen that several mutations in RBD (Q493R, Q498R, N501Y, G496S and S477N) contribute significantly to a high binding affinity with the human Angiotensin Converting Enzyme 2 (ACE2) receptor (6, 7). Interestingly, other mutations, however, cause this affinity to reduce considerably (K417N, Y505H and E484A). Therefore, not all the mutations in the Omicron variant help improve the affinity to the ACE2 receptor.

Additionally, most receptor-binding motif (RBM)-directed monoclonal antibodies lost *in vitro* neutralizing activity against Omicron (8). The Omicron substitutions have previously been found to independently reduce or even ablate antibody binding, and perhaps mediate antibody-mediated neutralization escape (9), raising concerns about the effectiveness of available vaccines and antibody therapeutics. Interestingly, although the neutralization of Omicron was undetectable in most subjects after vaccinations, individuals boosted with mRNA vaccines exhibited a potent neutralization of Omicron, only 4–6 times lower than wild type, suggesting enhanced cross-reactivity in the neutralizing antibody responses (10, 11).

Consequently, it seems necessary to clarify the role of the new Omicron variant in the interaction between the spike protein-ACE2 receptor and the immune evasion. Here, we hypothesize that the new conformation of the RBD in the spike of this variant does not back up what we know about its increased infectivity. To test the hypothesis, we carried out an *in-silico* study analyzing the structures of the SARS-CoV-2 spike protein, the protein electrostatic potential, the rest of the mutations found in Omicron spike protein, as well as the potential immune evasion of the changes.

## Methods

The analysis was based on two SARS-CoV-2 variants, the one isolated in Wuhan (hCoV-19/Wuhan/WH01/2019) here referred to as wild type (WT), and the first of the B.1.1.529 lineage detected in Botswana (Omicron; Table 1). The full-length genome sequences were downloaded from the Global Initiative on Sharing All Influenza Data (GISAID; https://www.gisaid.org/) and the protein sequence was obtained from the Research Collaboratory for Structural Bioinformatics Protein Data Bank (San Diego, CA). The translation of the peptide sequences from the nucleic acid sequences was estimated at the European Bioinformatics Institute's European Molecular Biology Laboratory using EMBOSS Transeq (12). One-letter notation of amino acid sequence was used (13).

**Table 1:** Details of the two SARS-CoV-2 variants.

	**Wuhan**	**B.1.1.529**
Virus name	hCoV-19/Wuhan/WH01/2019	hCoV-19 / Botswana / R40B60_BHP_3321001247/2021
Accession ID	EPI_ISL_406798	EPI_ISL_6640917
Type	Betacoronavirus	Betacoronavirus
GISAID Clade	L	GR
Lineage	B (Pango v.3.1.16 2021-11-18)	B.1.1.529 (Pango v.3.1.16 2021-11-18) B.1.1.529-like (Scorpio)
Location	Asia / China / Hubei / Wuhan	First detected in Botswana/Hong Kong/South Africa
Date	December 26th, 2019	November 11th, 2021
Variant		VUM GR/484A (B.1.1.529)

We used a multiple alignment of protein sequences software (Clustal Omega, Clustal, Dublin, Ireland) (14) arranging the sequences of DNA, RNA or protein to identify regions of similarity that may be a consequence of functional, structural, or evolutionary relationships between the sequences, and to construct an automatic multiple alignment of nucleotide or amino acid sequences (15, 16) between the two variants.

In order to compare the similarity between proteins, we used the “Ident and Sim” service in the Sequence Manipulation Suite, provided by the Universidad Complutense de Madrid, Spain (http://imed.med.ucm.es/Tools/SMS/ident_sim.html). From a group of aligned sequences (in FASTA or GDE format), this service calculates the identity and similarity of each sequence pair.

We used PyMOL to visualize and compare the molecules under study and produce images (17). The visualizer also enables us to make electrostatic calculations using the Adaptive Poisson-Boltzmann Solver plugin, whose program interface also permits us to visualize potential energy surfaces and charge densities on protein surfaces. Adaptive Poisson-Boltzmann Solver solves the equations of continuum electrostatics for large biomolecular assemblages. This software package was designed “from scratch” to ensure the integration with other computational packages and be improve as methods and applications change over time. We used this Adaptive Poisson-Boltzmann Solver plugin to predict protein interaction sites and also to map antigen epitopes (18). Using PyMOL, we can apply the mutation and visualize the interaction between the substituted residues in the spike protein of this lineage variant with the human ACE2 protein, according to a known structure, PDB ID 6m0j (19). The interactions resulting from these mutations with the cellular receptor were simulated and analyzed. The Adaptive Poisson-Boltzmann Solver complement of this application was also used to predict the sites of interaction between protein and spike epitopes.

The interaction of the spike proteins with the human ACE2 receptor was calculated for the WT variant and the Omicron variants using the open-source software PDBePISA. This is a web based interactive tool made available by the PDBe (Protein Data Bank in Europe, https://www.ebi.ac.uk/pdbe/; PISA: Proteins, Interfaces, Structures and Assemblies) to investigate the stability of formation of macromolecular complexes (protein, DNA/RNA and ligand) and to give detailed analysis of the surfaces, interfaces and assemblies between proteins (20). The following parameters were calculated with PDBePISA software: surface, which is the total solvent accessible surface area in square Angstroms (Å^2^); **Δ**^***i***^***G***, which indicates the solvation free energy gain upon formation of the interface, in kcal/mol - the value is calculated as the difference in total solvation energies of isolated and interfacing structures. That is why the values of Δ^i^G can be so low and even 0 in the case of non-interface residues (inaccessible residues or solvent-accessible residues). Therefore, the positive solvation energy Δ^i^G of a residue contributes negatively to the solvation energy gain of the interface, which corresponds to the hydrophobic effect. A negative Δ^i^G corresponds to hydrophobic interfaces, or positive protein affinity. Solvation energy estimates in PISA do not include the effect of satisfied hydrogen bonds and salt bridges across the interface; ***HSD***, which in the interface residues table indicates residues that contain the across-interface hydrogen bond, salt bridge or disulfide bond atoms. The corresponding table cells have a red background and contain letter H in case of hydrogen bond, S in case of salt bridge and D in case of disulfide bond or any combination of the above. A particular atom may be found from the Hydrogen Bond, Salt Bridge and Disulfide Bond Tables. The effect of hydrogen bonds (-0.44 kcal/mol per bond), salt bridges (additional −0.15 kcal/mol per salt bridge) and disulphide bonds (-4 kcal/mol per bond) is calculated separately; ***ASA***, which in the interface residues table indicates the solvent-accessible surface area of the corresponding residue, in Å^**2**^; and finally, ***BSA***, which in the interface residues table indicates the solvent-accessible surface area of the corresponding residue that is buried upon interface formation, in Å^**2**^. In this model, the buried area fraction is represented by a number of vertical bars that give a mnemonic representation showing bars which correspond to 10% of the total solvent-accessible surface area buried; iNat is the number of atoms of the interface; iNres is the number of residues of the interface.

### SARS-CoV-2 spike epitopes with sequence and immune evasion

In order to analyze the potential immune evasion of the viruses belonging to this lineage, we used the Immune Epitope Database (https://www.iedb.org/) information on 3,337 different epitopes in the spike protein, filtering *Organism* equals to *SARS-CoV-2 (ID: 2697049)* and *Antigen* equals to *spike glycoprotein (P0DTC2;* https://www.uniprot.org/uniprot/P0DTC2). In the epitope table obtained from the Immune Epitope Database, we filtered those epitopes that affect the RBD of the spike, to keep the epitopes involved in the potential neutralization of the coronavirus - that is, those that really matter in immunization - from position 317–533 of the glycoprotein. After filtering these epitopes, we looked for the sequences that included any of the substitutions existing in the Omicron variant and plotted this information constructing Immunome Browser maps.

### Sequence manipulation suite: Ident and sim

The Idem and Sim tool (http://imed.med.ucm.es/Tools/SMS/ident_sim.html) calculates the identity and similarity of aligned sequences (in FASTA or GDE format). Identity and similarity values are used to assess whether or not two sequences share a common ancestor or function.

## Results

### Alignment and change of physiochemical properties

**Figure 1:**
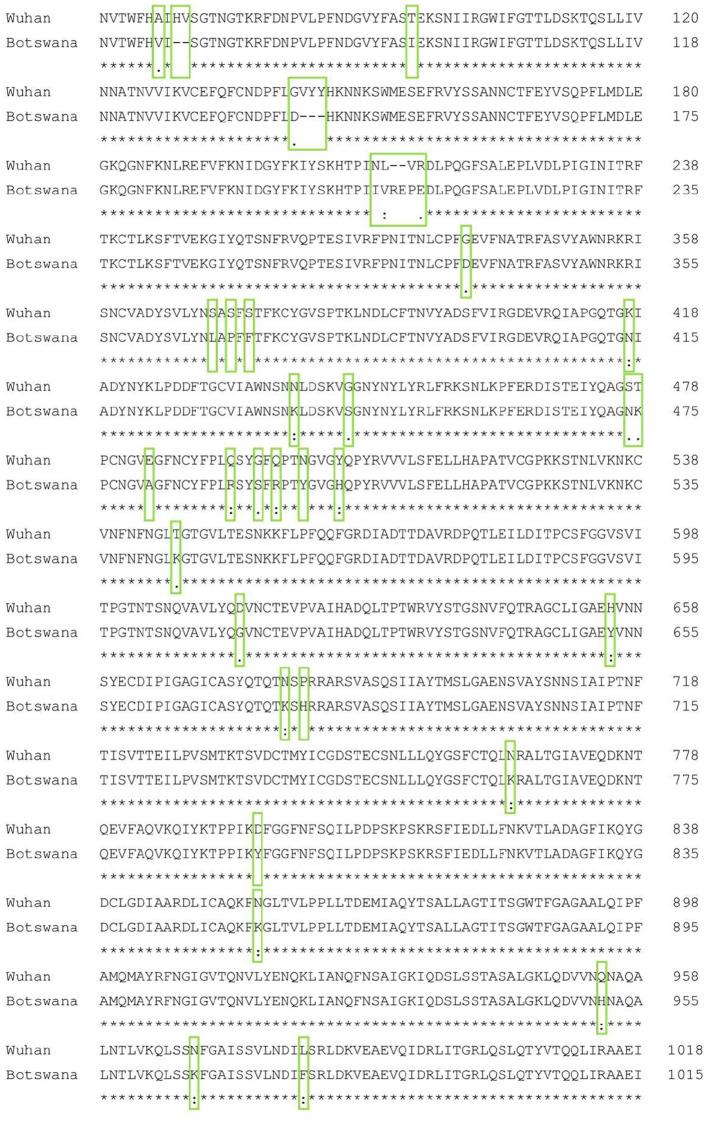
Spike protein amino acid sequence alignment of the Wuhan and Botswana variants. The differences between the two sequences are marked with a green box. A consensus line is shown below the nucleotide alignment of the two variants, with the following symbols indicating the degree of conservation observed for each compared pair: “^*^” (identical residues in all sequences), “:” (highly preserved column), “.” (weakly preserved column).

**Figure 2:**
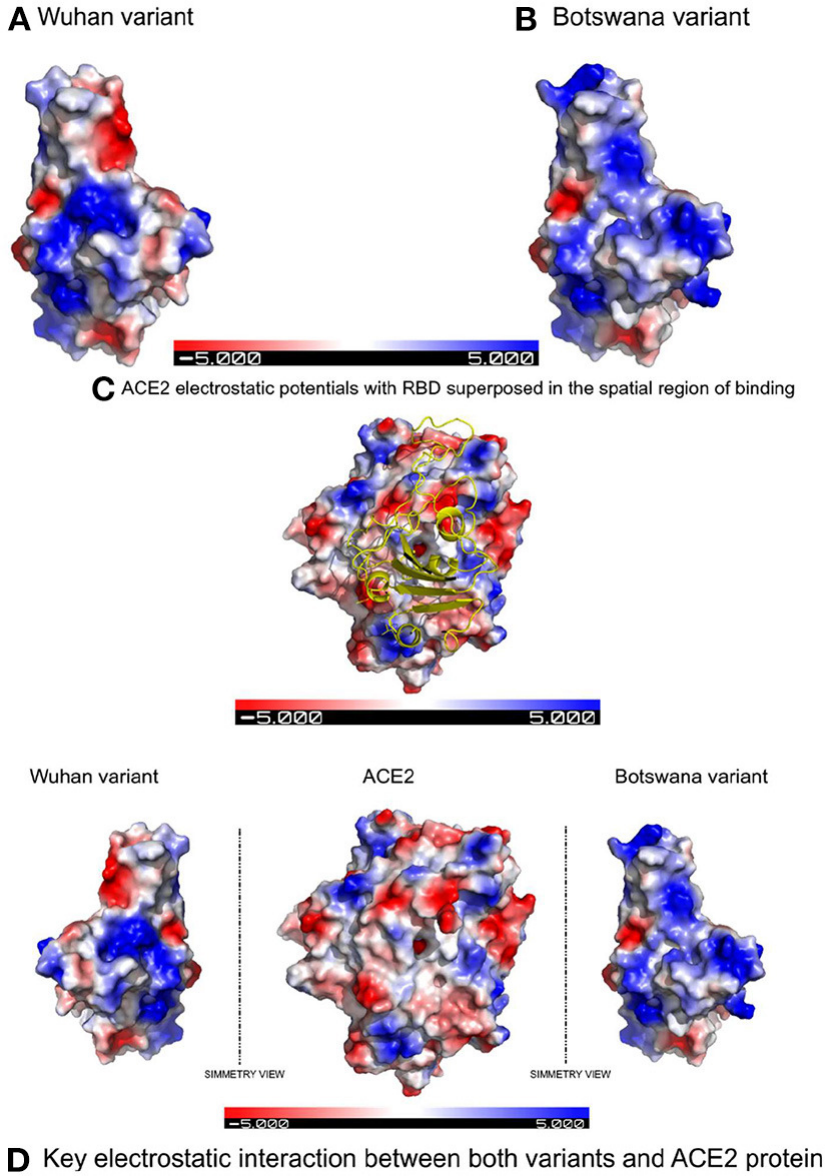
Electrostatic potentials comparative in the RBD between both variants and their influence in binding with ACE2 due to the latter's electrostatic potentials. **(A)** Electrostatic potentials in the wild-type RBD. **(B)** Electrostatic potentials in the RBD of the Botswana Omicron variant. **(C)** Representation of the spatial region showing of binding with the electrostatic potentials of the ACE2 protein superposed to the RBD of SARS-CoV-2 spike protein. **(D)** Symmetric comparation of the electrostatic potentials of both variants with de ACE2 protein.

Figure 1 shows the result of the translation of the aligned nucleotide sequences which encode the proteins of the two SARS-CoV-2 variants under study into their corresponding aminoacidic sequences. The new Botswana variant carries 61 amino acid changes with respect to the Wuhan variant (Table 2). The study of the spike protein resulted in 37 amino acid changes, 6 amino acid deletions, 1 insertion and 30 amino acid substitutions. Due to theses amino acid changes, the physicochemical properties of the spike changed and the electrostatic potentials differed for the RBD between both variants, as shown in Figure 2.

**Table 2:** List of amino acid changes between both variants.

Spike A67V Spike D614G Spike D796Y Spike E484A Spike G142D Spike G339D Spike G446S Spike G496S Spike H69del Spike H655Y Spike ins214EPE Spike K417N Spike L212I Spike L981F Spike N211del Spike N440K Spike N501Y Spike N679K Spike N764K Spike N856K Spike N969K	Spike P681H Spike Q493R Spike Q498R Spike Q954H Spike S371L Spike S373P Spike S375F Spike S477N Spike T95I Spike T478K Spike T547K Spike V70del Spike V143del Spike Y144del Spike Y145del Spike Y505H E T9I M A63T M D3G M Q19E N E31del	N G204R N P13L N R32del N R203K N S33del NSP3 A1892T NSP3 K38R NSP3 L1266I NSP3 S1265del NSP3 V1069I NSP4 T492I NSP5 P132H NSP6 G107del NSP6 I189V NSP6 L105del NSP6 S106del NSP12 P323L NSP14 I42V

NSP, Non-structural protein.

Table 3 includes only those residues that have suffered the mutation, and in this context, the solvation energy gain of the interface, Δ^i^G, bonded by these mutated residues is −3.6 kcal/mol, which corresponds to the hydrophobic effect and more protein-protein affinity, vs. 0 kcal/mol of Δ^i^G in the wild-type residues. However, if we consider the whole global interface of the interaction between ACE2 and RDB (Table 4), it is clear that the Δ^i^G of the wild-type Wu-01 RBD is −4.5 kcal/mol, compared with 0.8 kcal/mol of the B.1.1.529 RBD, which demonstrates that in global interactions of the complete interface between ACE2 and RBD, the best affinity is found on Wu-01 RBD. If we add the effect of hydrogen bonds (−0.44 kcal/mol per bond) and salt bridges (additional −0.15 kcal/mol per salt bridge) across the interface, it results in B.1.1.529 RBD having 13 hydrogen bonds and 1 salt bridge making a total of −5.87 kcal/mol of solvation energy, while Wu-01 RBD has 14 hydrogen bonds and 1 salt bridge which make −6.31 kcal/mol of solvation energy. The solvation free energy gain upon formation of the interface of ACE2 and Wu-01 RBD adds a further −0.44 kcal/mol, and it therefore shows a better protein-protein affinity.

**Table 3:** Key residue interactions with human ACE2 protein.

**Residue**	**Wu-01**					**B.1.1.529**				
	**HSDC**	**ASA**	**BSA**		**Δ^i^G**	**HSDC**	**ASA**	**BSA**		**Δ^i^G**
Spike K417N	HS	81.21	27.87	||||	−0.09		65.95	16.53	|||	−0.21
Spike N440K		122.89	0.00		0.00		193.44	0.00		0.00
Spike G446S	H	60.42	16.37	|||	0.01	H	79.15	9.09	||	0.01
Spike S477N		101.98	1.46	|	−0.02		149.11	4.87	|	0.05
Spike T478K		85.32	0.00		0.00		158.96	0.00		0.00
Spike E484A		110.71	13.62	||	−0.16		69.7	0.00		0.00
Spike Q493R	H	77.04	53.74	|||||||	0.01	HS	107.11	82.73	||||||||	−1.27
Spike G496S	H	28.35	22.54	||||||||	−0.04	H	60.23	50.87	|||||||||	−0.17
Spike Q498R		57.23	55.92	||||||||||	−0.30		108.35	108.03	||||||||||	−3.60
Spike N501Y	H	34.73	30.2	|||||||||	−0.10	H	38.05	32.22	||||||||	0.49
Spike Y505H	H	118.61	84.24	||||||||	0.69		93.24	59.52	|||||||	−0.03
				**Sum** **Δ** **^i^G** **=**	**0.00**				**Sum** **Δ** **^i^G** **=**	**−3.60**

In gray, solvent-accessible residues. ASA, accessible surface area in Å^2^. HSDC, residues making hydrogen, salt bridge, disulphide bond or covalent link. Δ^i^G, solvation energy effect, kcal/mol. BSA, buried surface area, Å^2^. The buried area percentage is expressed by the number of bars, one bar per 10%.

**Table 4:** Summary of global interactions between spike proteins and human ACE2 protein of both variants involve in the study.

**Variant**	**Human ACE2**			**RBD**			**Interface**	Δ^**i**^**G**	
	**iNat**	**iNres**	**Surface (Å^2^)**	**iNat**	**iNres**	**Surface (Å^2^)**	**Area (Å^2^)**	**kcal/mol**	***P*** **-value**
Wu-01	98	26	25,674	86	26	10,096	843.5	−4.5	0.513
B.1.1.529	101	25	25,681	87	24	10,447	827.6	0.8	0.761

### Interaction with ACE2 receptor

In addition to the physicochemical properties detailed above, there are several crucial changes in RBD surface electrostatic potentials, with a greater polarity and relevant change of exposed charge (to positive) in the Omicron variant. These two factors confirm the decrease in protein interactions in the RBD site of the new variant, due to an electrostatic repulsive force, when facing polarities of the same sign. The results of the *in-silico* study therefore show that mutations in the spike protein of the new variant of coronavirus do not establish a greater interaction with the ACE2 receptor compared to the primary lineage of the virus.

**Figure 3:**
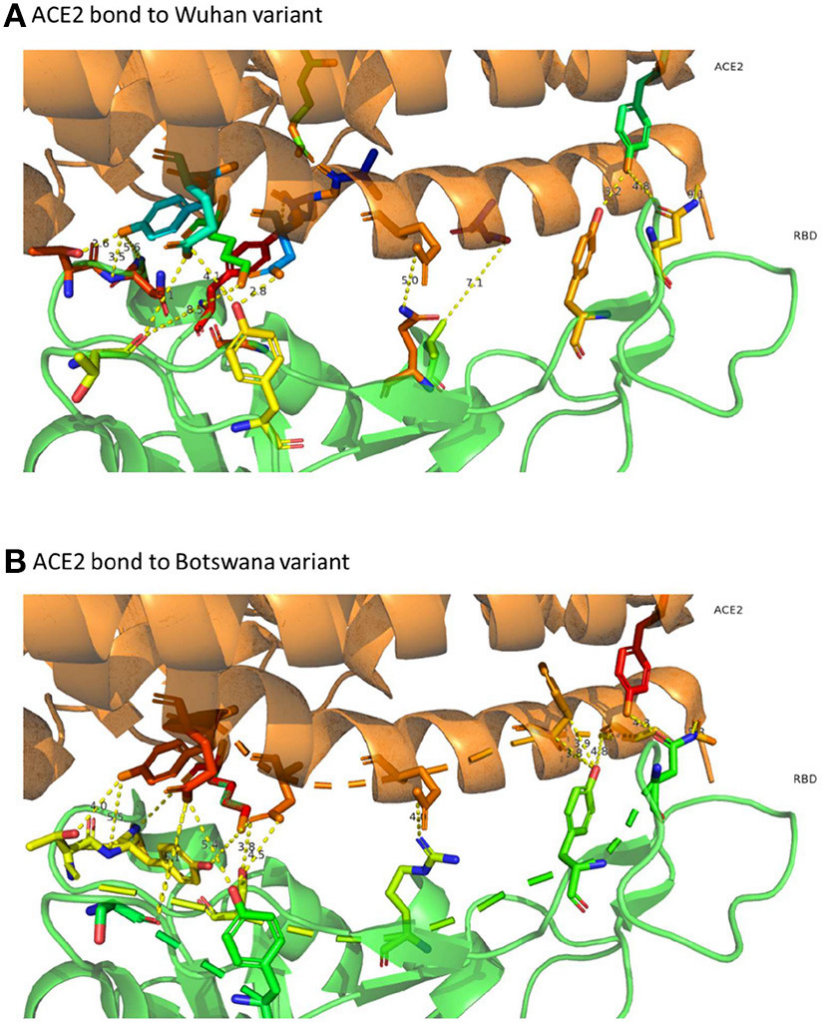
3D representation of ACE2 bonds to both variants. In **(A)** (ACE2 link to the Wuhan variant) it can be confirmed that the links are more numerous and with less distance than those that appear in **(B)** (ACE2 bond to Botswana variant). The yellow dashed line represents the distance between residues SARS-CoV-2 spike proteins and human ACE2 protein.

The distribution of hydrogen bonds and salt bridges of both variants is similar in structure and arrangement, as shown in Figure 3, which shows the capacity of ACE2 bond distribution represented after 3D modeling, for the two variants of SARS-CoV-2 under study. Based on protein docking studies, several mutations in RBD (Q493R, Q498R, N501Y, G496S and S477N) cause an alteration in the space between the amino acids of the ACE2-RBD interface, contributing significantly to a high binding affinity with the human ACE2 receptor, although other mutations reduce this affinity greatly (K417N, Y505H and E484A), with increased space in the ACE2-RBD interface and reduced affinity with human ACE2.

### Evasion of immunity

**Figure 4:**
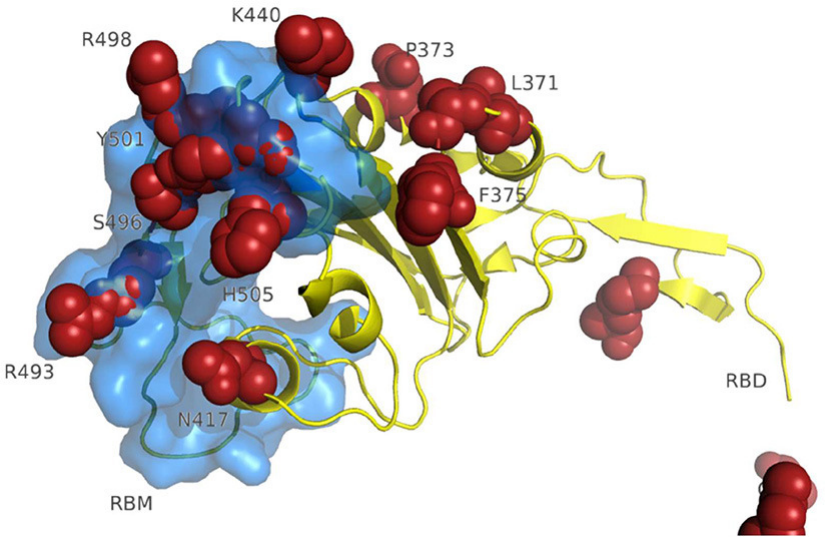
3D modeling of amino acid changes in the spike protein of the Omicron variant. Receptor-binding domain (yellow); RBM-receptor-binding motif (blue); B.1.1.529 mutations in red.

**Figure 5:**
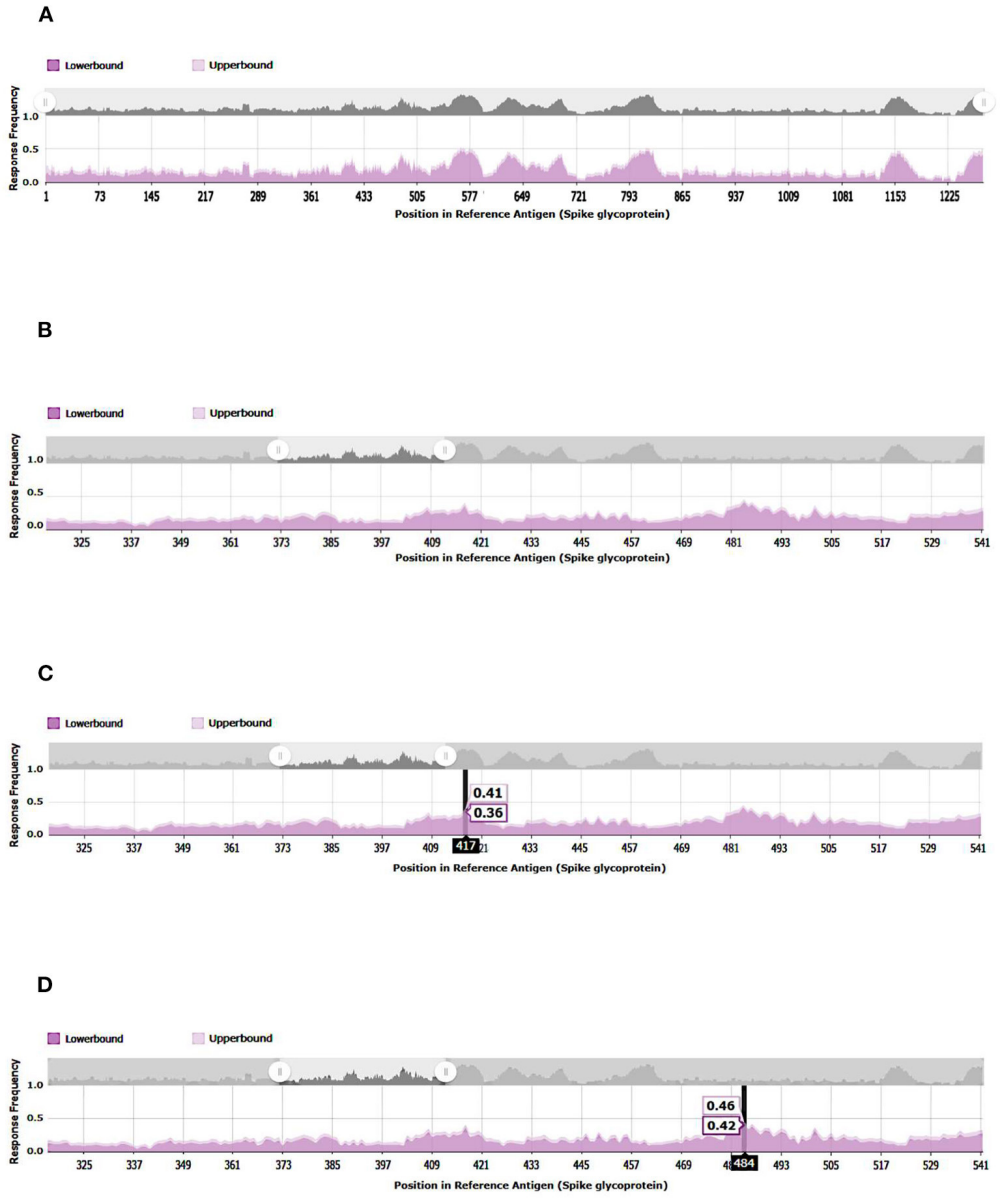
Immunome Browser maps, which shows the linear peptide epitopes filtered in the Immune Epitope Database along the SARS-CoV-2 glycoprotein spike sequence studied in immunoassays. **(A)** SARS-CoV2-S, spike glycoprotein (UniProt: P0DTC2). **(B)** SARS-CoV- 221 2-S-RBD, receptor-binding domain (UniProt: P0DTC2 amino acids 317 to 533). **(C)** Relevant amino acid of SARS-CoV-2-S-RBD involved in immune evasion. **(D)** Relevant amino acid of SARS-CoV-2-S-RBD involved in immune evasion.

A 3D modeling analysis can show the distributions of these new mutations between the RBD and the RBM, as shown in Figure 4. These changes compromise key RBM residues in the SARS-CoV-2 spike protein that interact with neutralizing antibodies and ACE2. In Figure 4, the mutations of the RBM region, the distal region of the spike protein, key in the neutralization of SARS-CoV-2, appear in red. The results of the epitopes that include any of the existing substitutions in the Botswana variant is shown in Table 1 in the online supplement and is plotted in the Immunome Browser maps in Figure 5, which shows the linear peptide epitopes filtered in the Immune Epitope Database along the SARS-CoV-2 glycoprotein spike sequence. The loss of neutralization is accounted in Figure 5, based on the frequency of the residues, substituted in the Botswana variant, that appear mapped in the epitopes of known neutralizing antibodies, matching the positions of the epitope with the positions of the residues in the SARS-CoV-2 spike protein. We can verify that some of the mutations of the new variant under study have a high frequency of appearance in the antigenic epitopes of previous variants. This graph allows us to explore how often each protein region has been studied in immunoassays. A total of 199 antigenic epitopes affected by the substituted/mutated amino acids have been located in this variant, out of a total of 3,337 (5.9%). Focusing on the RBD zone, we found that of the 958 known antigenic epitopes, 114 (11.9%) interact with neutralizing antibodies and, therefore, the substitutions/mutations, in the variant under study, affect the neutralizing function of these antibodies.

Applying the Ident and Sim tool to the RBDs of the SARS-CoV-2 variants under study and that of SARS-2005, we found, as expected, that if we calculate the identity between the three proteins, the proteins that share the greatest number of identical residues are higher among the variants of SARS-CoV-2. However, it was surprising to find that the homology of function measured by the similarity indicates that the RBD protein of the variant B.1.1.529 is closer to SARS-2005 than to the original Wu-01.

## Discussion

Our study details the epitopes compromised by the substitutions in the variant under study and in the regions of interest in the interaction between these proteins. We have shown that mutations and deletions in the SARS-CoV-2 spike protein of the variant under study, offer greater structural instability of that spike and change the affinity with the ACE2 receptor downward. In addition, we suggest the existence of other key receptors in the increase of infectivity. That is, the participation of other receptors in the increase of infectivity and the loss of neutralization capacity of antibodies generated in response to other variants. Similarly, the present report informs about the SARS-CoV-2 lineage under study, which has 6 nucleotides inserted with respect to the reference sequence and a gap of 36 nucleotides with respect to the primary lineage. These substitutions do not confer to this variant a higher capacity for interaction between the spike protein of the virus and the human ACE2 receptor, suggesting that the greater infectivity confirmed in clinical data must also be supported by other receptors and by the fact that it compromises residues that change the physiochemical properties of the protein-to-protein ligands, between host cells and the lineage under study. Additionally, RBM is key in the spike protein of SARS-CoV-2 which interacts with neutralizing antibodies. These modifications confer an ability to evade already acquired immunity against the new coronavirus in this new variant under study.

The first known confirmed B.1.1.529 infection was on November 9, 2021. This lineage is a clear example of the rapid molecular evolution of the new coronavirus, which has accumulated up to 60 mutations, 37 of which are located in the gene that encodes for the spike protein on the surface of the virus (21). Lineage B.1.1.529 represented a surprising evolution of SARS-CoV-2 for its molecular evolution. Up to that point, the virus had accumulated mutations at a rate of up to two nucleotides per month, but in the B.1.1.7 lineage, up to 19 nucleotide alterations were triggered, compared to the primary lineage, when isolated in January 2020.

Through bioinformatic applications, we found that the spike protein of the new viral variant B.1.1.529 does not establish a greater force of molecular interaction with the ACE2 receptor in human cells to which SARS-CoV-2 binds to make the infection viable, as has been hypothesized until now. Additionally, there are other mutations in the genome of the B.1.1.529 lineage that have not been previously analyzed which are involved in the difference in the pathological processes between the two variants under study. We have focused on the mutations involved in the binding of the spike protein to the ACE2 receptor, although the interactions that occur between the spike protein of SARS-CoV-2 and the cell membranes of the host cells also depend on the glycosylation of the virus protein. Protein-protein interactions are amplified due to the increased stability provided by glycans and their slip behavior, which positively affects the binding strength of these interactions (22). Previous studies have focused on the role of RBD mutations in the Omicron variant on the structure of the spike protein and its interactions with ACE2, but it has been shown through experiments that in cells that do not express ACE2 in their membrane, interactions, bonds and interactions do occur. Both electrostatic and hydrophobic interactions are responsible for the binding of SARS-CoV-2 with ACE2. Jawad et al. (23) show that this binding force is different in each variant and that it serves to explain the increase or decrease in infectivity of each one. Neverthelss, mutations and deletions can offer greater instability to S proteins, varying the strength and number of hydrogen bonds and, therefore, reducing the affinity and interaction with the ACE2 receptor, as reported by Casalino et al. (24). Furthermore, SARS-CoV-2 infects its victims through the participation of other non-ACE2 receptors (25). In the same line supporting our hypothesis, Gadanec at al. described other pathways leading to SARS-CoV-2 internalization stimulating infectivity without interaction with the ACE2 receptor (26).

The evasion of immunity in the Omicron variant, in which 12% of the antigenic epitopes are identified in the neutralization zone, suggests a loss of capacity in the antibodies generated, regardless of whether they are due to the vaccination process or by infection at any given time. Recent studies have suggested that low levels of antibody titers 6 months after vaccination do not provide sufficient antibodies to prevent infection by the Omicron variant (27). Thus, among individuals who have previously had COVID-19, a specific vaccination schedule may be required to induce detectable serum antibodies against the Omicron variant (28). Previous studies have shown that older patients exhibit a sustained SARS-CoV-2-specific antibody response 15 months after infection. This response multiplies the antibody response upon receipt of a single dose of vaccine after recovery from COVID-19. However, antibody responses in individuals who have not had the disease are multiplied only 6-fold after a second dose of vaccine (29). In a recent paper, the authors concluded that the Omicron-based recombinant protein vaccine elicited an altered serological response and exerted drastically reduced neutralizing activity against SARS-CoV-2, as well as a significantly weaker T-cell response (30). Recent work found increased odds of being infected with Omicron compared to other variants in the case of high virus copy number infections. Compared to unvaccinated individuals, the authors found a significant reduction in Omicron positivity rates against previous variants after several doses of the vaccine (31).

The combination of spike protein structure with antibody evasion may have contributed to its dominance over previous variants (32, 33). The clinical presentation of this Omicron variant has been described as considerably different from previous variants. Omicron results in less low respiratory tract involvement, and therefore appears to confer a lower likelihood of hospital admission. However, due to having a shorter period of illness and potentially higher infectivity, it is possible that the number of cases may increase, which may require special consideration to be given to occupational health policies and public health advice (34).

In conclusion, the scientific community has been alarmed by the potential immune evasion, increased infectivity and disease severity caused by the new variants of SARS-CoV-2, where the spike protein plays a crucial role in viral infectivity. These mutations appear to confer immune and enhanced infectivity properties, especially those linked to conformational changes in its structure. The vaccines appear to trigger a strong immune response to vaccination that can protect against most previous variants with multiple mutations in their sequence. The biggest risk of the Omicron variant is that it appears to be more effective at evading immune responses, largely due to numerous mutations in its spike protein.

## Data availability statement

The data supporting the findings of this study are available within the article. Derived data supporting the findings of this study are available from the corresponding author on request.

## Author contributions

JJ performed the *in-silico* model. CL and JL-C provided clinical insights. JL-C drafted the manuscript. All authors have read and agreed to the published version of the manuscript.

## Funding

This research was supported by CIBER-Consorcio Centro de Investigación Biomédica en Red- (CIBERES), Instituto de Salud Carlos III, Ministerio de Ciencia e Innovación.

## Conflict of interest

The authors declare that the research was conducted in the absence of any commercial or financial relationships that could be construed as a potential conflict of interest.

## Publisher's note

All claims expressed in this article are solely those of the authors and do not necessarily represent those of their affiliated organizations, or those of the publisher, the editors and the reviewers. Any product that may be evaluated in this article, or claim that may be made by its manufacturer, is not guaranteed or endorsed by the publisher.
