# Supplementary material for: Spike protein of SARS-CoV-2 Omicron variant: An in-silico study evaluating spike interactions and immune evasion
Source: Front Public Health. 2022 Nov 29;10:1052241. doi: 10.3389/fpubh.2022.1052241 (PMC9746896; doi:10.3389/fpubh.2022.1052241)
Supplement: Supplementary file 1 [file Table_1.DOCX]

Supplementary Material

Table 1. Complete list of epitomes.

| AA Substitution | Epitopes affected |
| --- | --- |
| Spike A67V | 1409235, 1539468, 1597653 |
| Spike H69del | 1318206, 1312678, 1448799, 1597477 |
| Spike V70del | 1528940 |
| Spike T95I | 1075071, 1513477, 1542714 |
| Spike G142D | 1312630, 1445629, 1597047, 1597702 |
| Spike V143del | 1531739, 1543331 |
| Spike Y144del | 1087268, 1309624, 1328953, 1334442, 1347908, 1347909, 1347914, 1347915, 1347694, 1391792, 1391793, 1397527, 1539041, 1597490 |
| Spike Y145del | 1087798, 1313994, 1391794, 1535090 |
| Spike N211del | 1071585, 1393954, 1392328, 1615090, 1482187 |
| Spike L212I | 1474503 |
| Spike ins214EPE | 1309567, 1496938 |
| Spike G339D | 1317333, 1540437, 1597629 |
| Spike S371L | 1310787, 1333450, 1392415, 1501918, 1594764 |
| Spike S373P | 1313549, 1391917 |
| Spike S375F | 1389151, 1542598 |
| Spike K417N | 1310238, 1087572, 1312824, 1319519, 1334674, 1334675, 1334676, 1334677, 1338780, 1347901, 1347902, 1346813, 1336258, 1393656, 1393657, 1338781, 1391999, 1346696, 1594753, 1594754, 1397519, 1539320, 1458911, 1597478, 1597230, 1597376, 1597719 |
| Spike N440K | 1075002, 1330538, 1581926, 1392000, 1334681, 1346819, 1481690 |
| Spike G446S | 1309117, 1087140, 1310037, 1310988, 1311243, 1314087, 1335923, 1335924, 1335925, 1336254, 1338779, 1347685, 1391788, 1397477, 1540464, 1397134, 1440442 |
| Spike S477N | 1313696, 1335944, 1335945, 1335946, 1335947, 1335948, 1394075, 1392570, 1397435, 1620718, 1510221 |
| Spike T478K | 1335952, 1517496 |
| Spike E484A | 1309459, 1314085, 1330444, 1330445, 1330446, 1334669, 1334670, 1334671, 1334672, 1334673, 1594766, 1336251, 1334684, 1334685, 1335914, 1335915, 1346812, 1393655, 1346693, 1346694, 1393743, 1397529, 1602300, 1427424, 1596867, 1597475, 1567301 |
| Spike Q493R | 1313385, 1398141, 1495380 |
| Spike G496S | 1310441, 1540449, 1439989 |
| Spike Q498R | 1394011, 1616502, 1494420 |
| Spike N501Y | 1310660, 1334682, 1338787, 1392324, 1397530, 1397521, 1397378, 1597481 |
| Spike Y505H | 1087809, 1314034, 1334394, 1394171 |
| Spike T547K | 1073424, 1311868, 1394095, 1621380 |
| Spike D614G | 1309454, 1566541, 1594765, 1379215, 1347684, 1625365 |
| Spike H655Y | 1070256, 1597476 |
| Spike N679K | 1483917, 1541827 |
| Spike P681H | 1310725, 1087687, 1392360, 1597483 |
| Spike N764K | 1393962, 1615277 |
| Spike D796Y | 1310327, 1309447, 1378524, 1597662 |
| Spike N856K | 1310659, 1309532 |
| Spike Q954H |  |
| Spike N969K | 1087650, 533050 |
| Spike L981F | 1310620, 1393912, 1392288, 1613822, 1613823, 1472882 |
